# La tuberculose pulmonaire et le tabac: à propos de 100 cas

**DOI:** 10.11604/pamj.2014.19.202.5329

**Published:** 2014-10-24

**Authors:** Hicham Janah, Hicham Souhi, Hatim Kouissmi, Karima Marc, Rachida Zahraoui, Jouda Benamor, Mona Soualhi, Jamal Eddine Bourkadi

**Affiliations:** 1 Service de Phtisiologie de l'Hôpital Moulay Youssef, CHU Rabat, Maroc

**Keywords:** Tuberculose pulmonaire, tabagisme, charge bacillaire, pulmonary tuberculosis, smoking, bacillary load

## Abstract

Le tabagisme et la tuberculose sont deux enjeux majeurs de santé publique au niveau mondial, en particulier dans les pays émergents. Pour déterminer les particularités cliniques, radiologiques, bactériologiques et thérapeutiques de la tuberculose pulmonaire chez les sujets tabagiques nous avons mené une étude prospective au service de phtisiologie de l'hôpital Moulay Youssef sur une période de 10 mois, portant sur 100 nouveaux cas de tuberculose pulmonaire, répartis en 2 groupes, 50 patients tabagiques: Groupe A et 50 patients non tabagiques: Groupe B. Tous nos patients étaient de sexe masculin, l’âge moyen était de 41 ans ± 12 chez le groupe A et de 36 ans ± 16 chez le groupe B. Le délai de consultation était plus long chez les tabagiques, la médiane était de 60j (30; 98) contre 40j (30; 60), la symptomatologie clinique était variable chez les deux groupes, dominée par les expectorations chez les tabagiques 96% contre 60%. Les lésions radiologiques étaient similaires chez les deux groupes ainsi que la charge bacillaire. Tous les patients ont été mis sous traitement antituberculeux. Après un mois du traitement, la Bacilloscopie était négative chez 50% du groupe A contre 66% chez le groupe B. la régression des lésions radiologiques était similaire chez les deux groupes. Le retard diagnostique et le retard de négativation des frottis sont les principales particularités de la tuberculose pulmonaire du sujet tabagique. Le sevrage tabagique doit faire partie intégrante de la prise en charge des patients atteints de tuberculose.

## Introduction

Le tabagisme et la tuberculose sont deux enjeux majeurs de santé publique au niveau mondial, en particulier dans les pays émergents. La relation entre le tabac et la tuberculose pulmonaire a été suspectée depuis 1918 et ce n'est que récemment que l'effet du tabac sur la tuberculose a été identifié [1]. Le but de notre étude est d'apporter des éléments sur l'effet du tabagisme sur les aspects cliniques, bactériologiques, radiologiques et évolutifs de la tuberculose pulmonaire.

## Méthodes

Etude prospective menée au service de phtisiologie de l'hôpital universitaire Moulay Youssef de Rabat sur une période de 10 mois, portant sur 100 nouveaux cas hospitalisés pour tuberculose pulmonaire à microscopie positive, répartis en 2 groupes, 50 patients tabagiques: Groupe A et 50 patients non tabagiques: Groupe B. Ont été exclus de ce travail les patients immunodéprimés.

## Résultats

**Figure 1:**
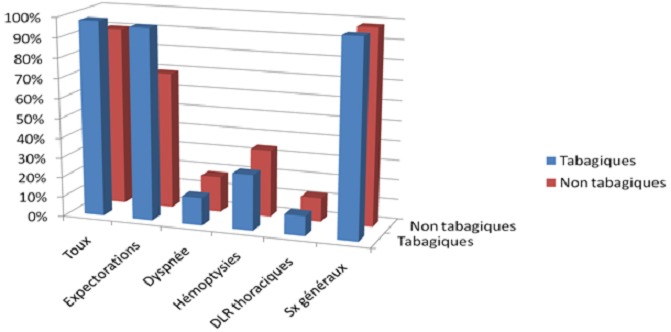
Comparaison des symptômes cliniques chez les deux groupes

**Figure 2:**
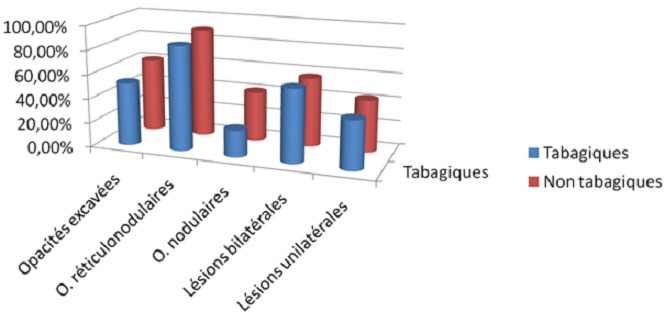
Comparaison des anomalies radiologiques observées chez les deux groupes

**Figure 3:**
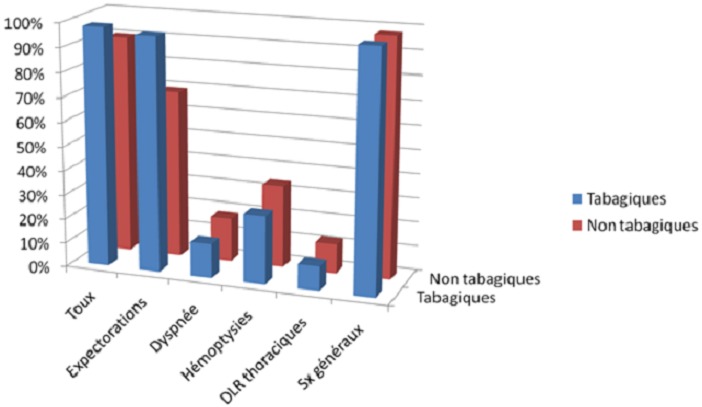
Importance de la charge bacillaire chez les deux groupes

L’âge moyen était de 41 ans ± 12 chez le groupe A et de 36 ans ± 16 chez le groupe B. Dans le groupe A, le degré d'intoxication tabagique variait de 10 à 80 paquets/année (PA) avec un degré moyen d'intoxication estimé à 18 paquets/année. Le délai de consultation était plus long chez les tabagiques, la médiane était de 60j (30; 98) contre 40j (30; 60). La symptomatologie clinique était variable chez les deux groupes, dominée par les expectorations chez les tabagiques: 96% contre 60% (Figure 1). Les lésions radiologiques étaient similaires chez les deux groupes (Figure 2) ainsi que la charge bacillaire (Figure 3). Le traitement était similaire dans les 2 groupes associant rifampicine - isoniazide - pyrazynamide et streptomycine ou éthambutol pendant 2 mois puis rifampicine et isoniazide pendant 4 mois. Après un mois du traitement, la Bacilloscopie était négative chez 50% du groupe A contre 66% chez le groupe B. Nous n'avons relevé de mauvaise observance du traitement significative chez les deux groupes. Le traitement était globalement bien toléré chez les deux groupes. La régression des lésions radiologiques était similaire chez les deux groupes.

## Discussion

Le tabagisme et la tuberculose sont deux enjeux majeurs de santé publique au niveau mondial, en particulier dans les pays émergents. La fumée de tabac favorise l'infection à Mycobacterium tuberculosis par plusieurs mécanismes: altération de la clairance muco-cilaire [1, 2], diminution des performances des macrophages alvéolaires [3, 4], immunodépression des lymphocytes pulmonaires [5], diminution de l'activité cytotoxique des cellules natural killer, altération de l'activité des cellules dendritiques pulmonaires [6–8]. La promiscuité, le bas niveau socio-économique, l'infection par le VIH et la susceptibilité génétique à la tuberculose [9, 10] sont des facteurs qui contribuent à entretenir la tuberculose. Le tabagisme constitue l'un des facteurs de risque favorisant la survenue de cette maladie. Davies et al. ont montré que l'incidence de la tuberculose augmentait avec la consommation du tabac, et que ce risque était multiplié par 4 à partir d'une consommation supérieure à 20 cigarettes par jour [11].

Concernant l’étude de la symptomatologie clinique, nous n'avons trouvé de différence entre les 2 groupes que dans les expectorations. L’étude de Fekih et al [12] a montré Il n'y avait pas de différence significative entre fumeurs et non-fumeurs concernant les signes cliniques de la maladie (toux et amaigrissement). Contrairement à d'autres études qui ont montré que les signes cliniques au cours de la tuberculose étaient beaucoup plus importants en cas de tabagisme associé [13]. Le délai de consultation est plus chez les tabagiques. L’étude de Fekih et al. [12] a montré aussi un retard diagnostique de la tuberculose pulmonaire supérieur chez les fumeurs par rapport aux non-fumeurs

Sur le plan Radiologique, nous avons trouvé une association de lésions (nodules, infiltrats et excavations) chez les deux groupes. Dans une étude réalisée en Tunisie [14], la sévérité des lésions radiologiques initiales (nodules, infiltrats, opacités excavées) et les séquelles radiologiques (opacités excavées et/ou fibrose pulmonaire) de tuberculose pulmonaire étaient plus importantes chez les fumeurs que chez les non-fumeurs. D'autre part, l’étude de Fekih et al [12] a montré les lésions radiologiques initiales (bilatéralité), étaient plus importantes et fréquentes chez les fumeurs que chez les non-fumeurs. Il en était de même pour les séquelles cliniques (dyspnée) et radiologiques (fibrose pulmonaire). Plusieurs études ont montrés que Le tabagisme était associé à une mauvaise observance du traitement [15–19].

Comme dans notre, Un délai plus prolongé de la négativation des bascilloscopies a été observé pour les tabagiques comparé au groupe non tabagiques dans d'autres séries [14, 20]. Ainsi l’étude de Fekih et al. [12] a montré un délai de guérison de la tuberculose pulmonaire supérieur chez les fumeurs par rapport aux non-fumeurs L'OMS [21] a rappelé les recommandations des experts: l'arrêt du tabagisme est un moyen essentiel de contrôle de la TB dans les pays émergents [22, 23]. Des procédures d'aide à l'arrêt du tabagisme ont été élaborées afin de faciliter le soutien apporté aux patients fumeurs lors du suivi de leur tuberculose maladie. La stratégie DOTS peut se prêter à un accompagnement de l'arrêt du tabagisme [24].

## Conclusion

Le retard diagnostique et le retard de négativation des frottis sont les principales particularités de la tuberculose pulmonaire du sujet tabagique. La fumée de tabac, en altérant les défenses du poumon contre l'infection par Mycobacterium tuberculosis, est un des facteurs augmentant le risque de tuberculose. Le sevrage tabagique doit faire partie intégrante de la prise en charge des patients atteints de tuberculose.
